# A non-inheritable maternal Cas9-based multiple-gene editing system in mice

**DOI:** 10.1038/srep20011

**Published:** 2016-01-28

**Authors:** Takayuki Sakurai, Akiko Kamiyoshi, Hisaka Kawate, Chie Mori, Satoshi Watanabe, Megumu Tanaka, Ryuichi Uetake, Masahiro Sato, Takayuki Shindo

**Affiliations:** 1 Department of Cardiovascular Research, Graduate School of Medicine, Shinshu University, 3-1-1 Asahi, Matsumoto, Nagano 390-8621, Japan; 2 Animal Genome Research Unit, Division of Animal Science, National Institute of Agrobiological Sciences, 2-1-2 Kannondai, Tsukuba, Ibaraki 305-8602, Japan; 3 Section of Gene Expression Regulation, Frontier Science Research Center, Kagoshima University, 8-35-1 Sakuragaoka, Kagoshima, Kagoshima 890-8544, Japan

## Abstract

The CRISPR/Cas9 system is capable of editing multiple genes through one-step zygote injection. The preexisting method is largely based on the co-injection of *Cas9* DNA (or mRNA) and guide RNAs (gRNAs); however, it is unclear how many genes can be simultaneously edited by this method, and a reliable means to generate transgenic (Tg) animals with multiple gene editing has yet to be developed. Here, we employed non-inheritable maternal Cas9 (maCas9) protein derived from Tg mice with systemic Cas9 overexpression (Cas9 mice). The maCas9 protein in zygotes derived from mating or *in vitro* fertilization of Tg/+ oocytes and +/+ sperm could successfully edit the target genome. The efficiency of such maCas9-based genome editing was comparable to that of zygote microinjection–based genome editing widely used at present. Furthermore, we demonstrated a novel approach to create “*Cas9* transgene-free” gene-modified mice using non-Tg (+/+) zygotes carrying maCas9. The maCas9 protein in mouse zygotes edited nine target loci simultaneously after injection with nine different gRNAs alone. Cas9 mouse-derived zygotes have the potential to facilitate the creation of genetically modified animals carrying the *Cas9* transgene, enabling repeatable genome engineering and the production of *Cas9* transgene-free mice.

Extensive research has been dedicated to understanding the intricacies of the genome and its modifications, and has furthered development in the medical and agricultural fields. For the last decade, a series of revolutionary genome modification technologies—including ZFN (zinc finger nuclease), TALEN (transcriptional activator-like effector nucleases), and CRISPR/Cas9 (clustered regularly interspaced short palindromic repeat/CRISPR-associated protein 9) systems—have proven useful for the genetic knockout (KO) of targeted loci^1^,^2^,^3^,^4^,^5^. Among these three methods, CRISPR/Cas9 has become readily used in many laboratories for genome editing because of its convenient vector design^6^,^7^. The CRISPR/Cas9 system consists of a Cas9 endonuclease and guide RNA (gRNA), the latter of which is comprised of a 20-nucleotide complementary to the genomic site(s) of interest and a NGG protospacer-adjacent motif (PAM) that directs the Cas9 to induce targeted DNA double-stranded breaks (DSBs)^6^. These Cas9-mediated DSBs are then repaired through either non-homologous end joining (NHEJ) or homologous recombination (HR). Importantly, while NHEJ results in an insertion and deletion (indel) mutation that can cause frameshift wild-type reading frame, HR-mediated repair can lead to the incorporation of a homologous fragment when it present around the DSB sequences.

CRISPR/Cas9 has simplified the process of generating genetically engineered animals^8^,^9^,^10^,^11^,^12^,^13^,^14^,^15^, which is usually accomplished using HR-based traditional gene targeting methods in mouse embryonic stem (ES) cells^16^. Notably, the CRISPR/Cas9 system overcomes the need to obtain ES cells or specific mouse strains customized for chimera formation. Moreover, CRISPR/Cas9 also allows users to edit multiple loci in a cell through one-shot gene delivery using two or more gRNAs^6^,^7^.

Presently, many CRISPR/Cas9-based genome-edited animals are produced by the one-step microinjection of Cas9 DNA (or mRNA) and a gRNA expression vector (or gRNA) into a zygote^17^,^18^,^19^. However, this strategy may restrict the simultaneous mutation induction of multiple target loci, since the presence of Cas9 DNA (or mRNA) can interfere with increased solution containing multiple gRNAs, due to the limited capacities of the pronucleus and cytoplasm. An ideal approach to overcome this problem would be the creation of Cas9-expresing cells or organisms, which would only require the introduction of gRNA(s) to induce mutations at target loci. Unfortunately, it remains unknown whether Cas9 overexpression affects the normal function of organisms, when we started this project. Recently, Platt *et al*.^20^ produced a conditional Cas9-expressing mouse line, in which the transgenes had been knocked-in in the *Rosa26* locus on chromosome 6, and indicated that this line is phenotypically normal and fertile.

In this study, we generated transgenic (Tg) mice with multiple transgene copies exhibiting systemic Cas9 overexpression (hereafter referred to as “Cas9 mice”) and assessed its safety and efficacy in targeted genome editing *in vitro* and *in vivo*. Interestingly, we found that non-inheritable maternal Cas9 (maCas9) existing in zygotes had the ability to edit multiple target loci simultaneously in the absence of Cas9 transgene.

## Results

### The Cas9 mice exhibit genetically, morphologically and physiologically normal properties

**Figure 1:**
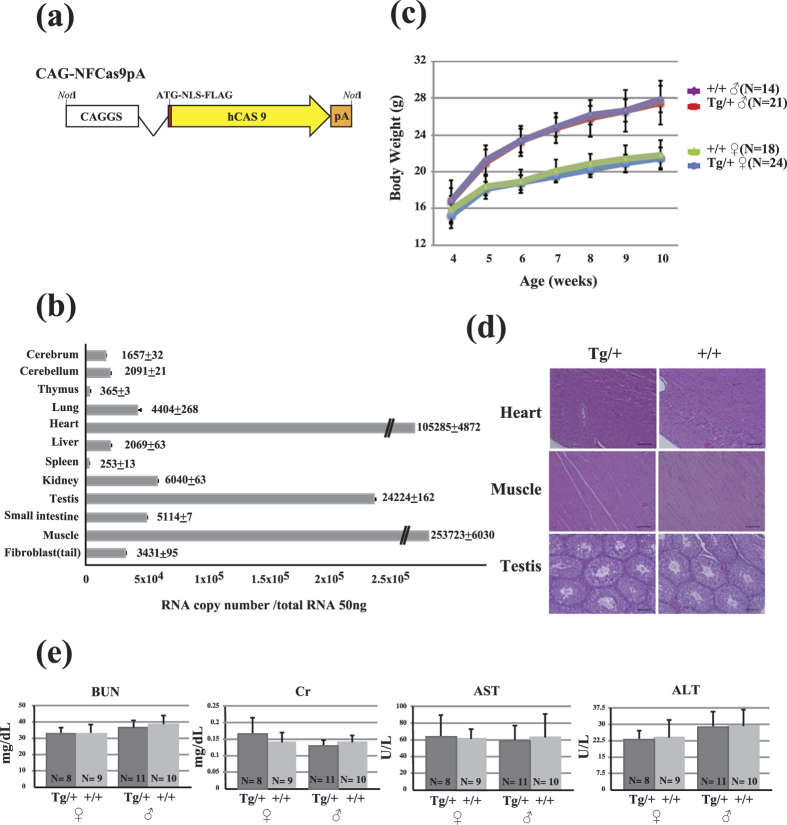
Characterization of a transgenic (Tg) mouse line (NFCas9-2) expressing humanized Cas9 systemically. (**a**) Schematic of the NFCas9 transgene construct, which confers ubiquitous *Cas9* expression under the chicken β-actin promoter, CAG. N and F indicate the SV40-derived nuclear location signal (NLS) and FLAG sequences, respectively. (**b**) Quantitation of *Cas9* mRNA expression in the major organs. Relative *Cas9* mRNA copy number was determined by real-time qPCR. (**c**) Body weight change of both male and female mice aged 4 to 10 weeks. (**d**) Organs were collected from 10-week-old Cas9/+ and +/+ mice (n = 3 each). Representative images are shown. Scale bar = 100 μm. (**e**) Evaluation of serum levels of blood urea nitrogen (BUN), creatinine (Cr), aspartate aminotransferase (AST), and alanine aminotransferase (ALT) in 10-week-old Tg mice (Cas9/+ female, n = 8; Cas9/+ male, n = 11) and their non-Tg littermates (+/+ male, n = 9; +/+ female, n = 10). Data are represented as the mean ± standard deviation (SD).

We produced a total of nine Cas9 mouse lines, four carrying NFCas9 and five FCas9, which contain the SV40 nuclear localization signal (NLS) and FLAG tag or FLAG alone, respectively (Fig. 1 and Fig. S1). The production efficiency of Cas9 mice was similar to that of Tg mice previously reported^21^, suggesting that constitutive Cas9 expression had conveyed no ill effects on mice from the zygote to weaning stage. Half of the NFCas9 and all five FCas9 founder mice passed their transgenes onto offspring (Fig. S2a). Quantitative (q)RT-PCR, RT-PCR, and western blot analyses demonstrated Cas9 expression in the tail-derived fibroblasts using F1 offspring of these seven Tg lines (Figs S2b and S2d). Based on these data, the NFCas9-2 line with strong Cas9 expression was selected for use in further analyses. This line carried nine transgene copies per diploid cell (Figs S2a and S2c)] that integrated into the *Gtpbp10* gene (Fig. S3), and exhibited a simple Mendelian inheritance pattern (Tables S1). The homozygous mice of this line were viable (Tables S2), and the average number of pups born was similar to that of the BDF1 strain (6 pups/litter). Moreover, qRT-PCR analysis revealed a ubiquitous expression of Cas9 mRNA, which was strongest in the heart, skeletal muscle, and testis (Fig. 1b and Fig. S2b). Histological analysis revealed no overt morphological abnormalities (Fig. 1d and Fig. S4), and no notable differences in growth rate or physiological maker values—including blood urea nitrogen (BUN), creatinine (Cr), aspartate aminotransferase (AST), and alanine aminotransferase (ALT)—were observed between Tg and non-Tg littermates (Fig. 1e).

### Wild-type zygotes derived from Cas9 mice possess a high genome editing activity, probably due to the presence of maCas9

**Figure 2:**
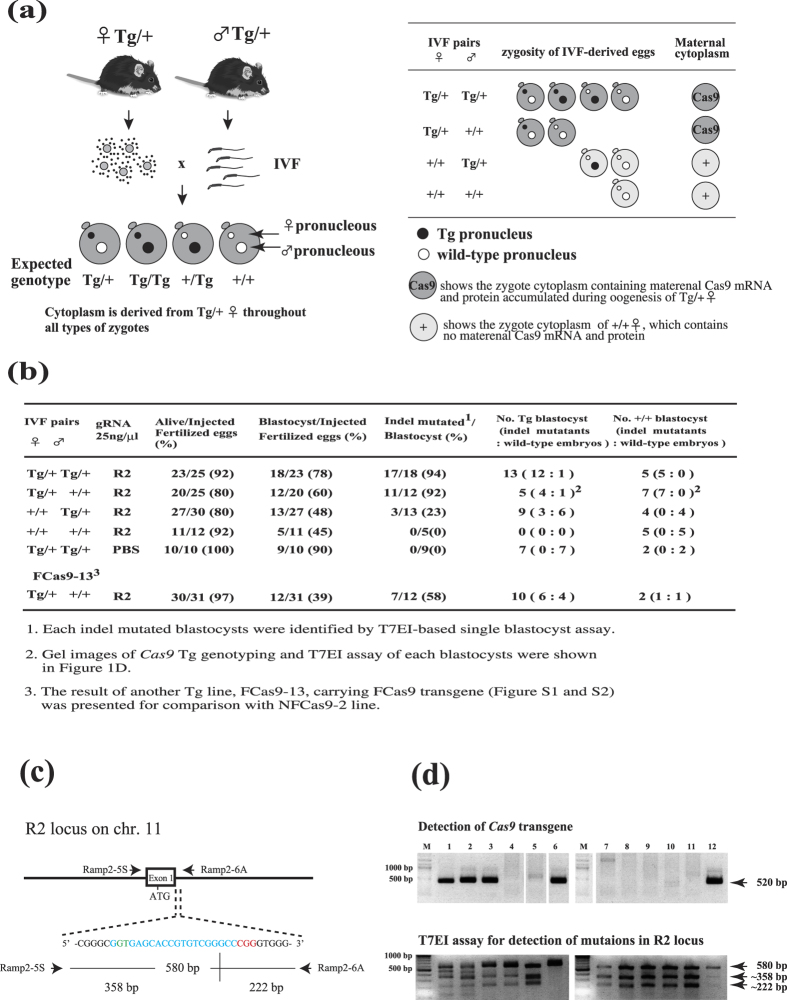
Efficiency of indel mutations mediated by constitutive Cas9 expression in several IVF pairs, and creation of a Cas9 transgene-free gene-modified mouse using non-inheritable maternal Cas9-containing zygotes. (**a**) Left panel: Schematic illustration of the generation of variable types of zygotes after IVF with different combinations of oocytes and sperm. Right panel: Expected Cas9 transgene genotypic frequencies of zygotes and maternal cytoplasm pattern. The original mice were drawn by Dr. Akira Imai (A.I.), our laboratory member of Shinshu University. (**b**) CRISPR/Cas9-mediated Ramp2 (R2) gene mutations in the offspring (blastocysts) of gRNA-injected zygotes obtained by different IVF combinations in the NFCas9-2 line. (**c**) Schematic of R2-gRNA-mediated genome editing at the 15th nucleotide region from the 3′ end of exon 1 in the murine *Ramp2* gene. The R2-gRNA-coding sequence is shown in blue. The donor splice site (GT) is shown in green. The protospacer-adjacent motif (PAM) sequence is shown in red. (**d**) Gel images of *Cas9* genotyping and T7EI assay for each blastocyst developed from IVF between a Tg/+ female and +/+ male, shown in (**b**) and subsequent microinjection with R2 gRNA alone.

We considered that zygotes derived from Cas9 mice might possess Cas9 mRNA (and/or protein) as a maternally inherited factor (hereafter referred to as maCas9), in which case there would be no need for the Cas9 transgene. To assess this, we injected R2-gRNAs directed to the *Ramp2* gene alone into the pronucleus of zygotes obtained from *in vitro* fertilization (IVF) with various breeding pairs (Fig. 2a,b). The resulting single blastocyst was then subjected to both Cas9 Tg genotyping and a T7 endonuclease I (T7EI)-based assay (Fig. 2c,d). The T7EI assay revealed high rates (92–94%) of indel mutations when IVF was performed between heterozygous transgenics (Tg/+) or between Tg/+ and wild-type (+/+) mice, indicating that the existing Cas9 in the Tg zygotes has a high degree of genome editing activity. Notably, high rates of indel mutations were also observed when Tg/+ oocytes were fertilized with Tg/+ or +/+ sperm. In contrast, +/+ oocytes fertilized with Tg/+ sperm exhibited a low mutation rate (23%). In addition, we examined the relationship between blastocyst genotype and genome editing activity and found that “Cas9-transgene-free” blastocysts have a high rate of indel mutation (Fig. 2b). For example, all +/+ blastocysts examined harbored mutations in R2 locus (5+7/5+7) when IVF was performed between Tg/+ pairs or Tg/+ and +/+ mice. This suggests that maCas9 mRNA (and/or protein) should be the primary means of genome editing in zygotes, since zygotic gene activation is absent at this stage and transcripts including Cas9 are gradually translated into proteins until the 2-cell stage^22^. We also observed this phenomenon with another Tg line, FCas9-13 (Fig. 2b).

Notably, genome editing in zygotes by gRNA injection alone was specific to the target locus (Fig. S5). For example, when zygotes were injected with R1-gRNA alone, indel mutations were limited to the *Ramp1* locus. Similar results were obtained when zygotes were injected only with R2-gRNA. Correspondingly, injection of both R1- and R2-gRNAs resulted in the simultaneous induction of indel mutations at both loci. When R1- and R2-gRNA were co-injected into Tg zygotes (Tg/+ × +/+) and their offspring (blastocysts and fetuses) were inspected for the presence of indel mutations, almost all were heterozygous for the indel mutations; however, all others displayed double KO phenotypes (Fig. S6). Three potential off-target sites for each locus (*Ramp1* and *Ramp2* genes) were analyzed using 10 fetal samples obtained (Experiment 1 of Fig. S6). No appreciable off-target mutation was identified for these two genes (Table S7).

### The efficiency of maCas9-based genome editing is comparable to that of zygote microinjection-based genome editing

**Figure 3:**
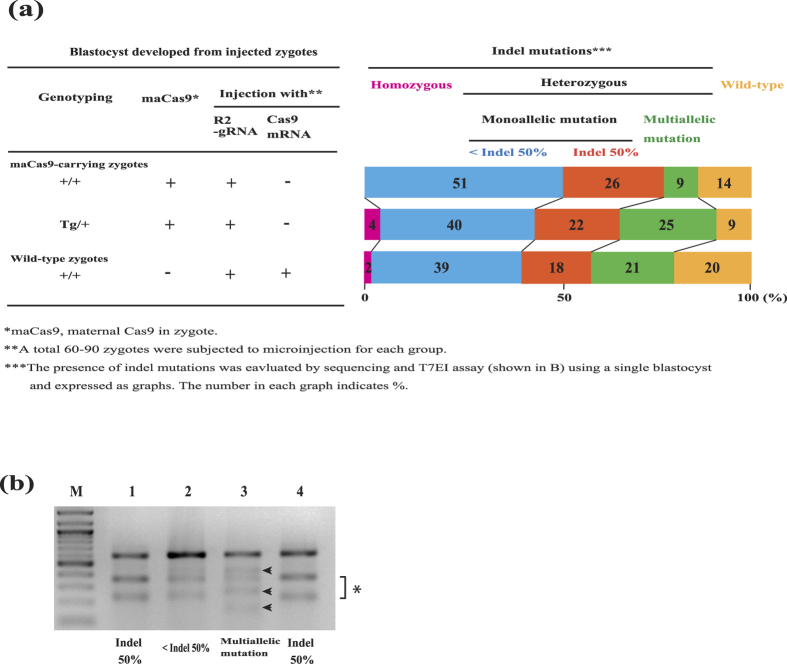
Characterization of maCas9-induced zygotic genome editing. (**a**) The rates of mutation induction and the state of indel mutations, namely homozygous (biallelic) or heterozygous (monoallelic or multiallelic) KO of a target gene in maCas9-carrying zygotes (+/+ and Tg/+) and wild-type zygotes (+/+). There was no significant difference (*p* > 0.05, one way-ANOVA) in the number of zygotes with homozygous mutation or zygotes with heterozygous mutation among the three genetic groups. A total of 35–42 blastocysts for each group were tested for possible mutations by sequencing (data not shown) and the T7E1-based assay shown in (**b**). “<Indel 50%” indicates samples in which the total intensity of two cleaved bands is less than that of the parental band, as shown in lane 2 in (**b**). “Indel 50%” indicates samples in which the total intensity of two cleaved bands is nearly equivalent to that of the parental band, as shown in lanes 1 and 4 in (**b**). Multiallelic mutation indicates samples exhibiting more than two cleaved bands generated after the T7E1 assay, as shown in lane 3 in (**b**). Experiments were repeated 3 times. (**b**) A representative gel image of T7EI-treated PCR products derived from 3 individual blastocysts (lanes 1–3). Lane 4: 50% indel control (monoallelic and no-mosaicism), which was used to determine whether these blastocysts (lanes 1–3) have mosaic indel mutations. *indicates the two expected cleaved bands after T7E1-mediated digestion. Arrowheads in lane 3 indicate another band besides the two expected cleaved bands, showing multiallelic mutation. M: lambda DNA digested with *Hin*dIII + 100-bp ladder markers.

In this study, we first demonstrated that maCas9 is useful for editing the zygotic genome. However, the precise efficiency for genome editing in mice using this novel system remains unknown. Therefore, we attempted to compare our maCas9-based genome editing system with the previously established genome editing method in zygotes based on the microinjection of exogenous *Cas9* mRNA and gRNA. Zygotes carrying maCas9 were prepared by IVF of Tg/+ oocytes and +/+ sperm. Wild-type (+/+) zygotes were prepared by IVF of +/+ oocytes and +/+ sperm (Fig. 3). R2-gRNA (25 ng/μl) alone was microinjected into both the cytoplasm and pronuclei of zygotes carrying maCas9 (Tg/+ or +/+), whereas both synthesized Cas9mRNA (50 ng/μl) and R2-gRNA (25 ng/μl) were injected into both the cytoplasm and pronuclei of wild-type (+/+) zygotes. The resulting single blastocyst was then subjected to both *Cas9* Tg genotyping and sequencing of the target alleles of *Ramp2* (Fig. 3a). Furthermore, the blastocysts that had been judged as those with heterozygous (monoallelic or multiallelic) mutations were subjected to a T7EI-based assay (Fig. 3a,b). The results demonstrated that the rate of mutation induction and the state of indel mutations, namely homozygous (biallelic), monoallelic, or multiallelic KO of a target gene, appeared to be statistically similar among the three different genetic groups (+/+ and Tg/+ zygotes for maCas9-based genome editing and +/+ zygotes for zygote microinjection–based genome editing) (*p* > 0.05, one way-ANOVA; Fig. 3a). Notably, the rates of non-mosaic (50% indel mutation) and mosaic (<50% monoallelic indel mutation and multiallelic mutation) mutations in the maCas9-containing zygotes (+/+ and Tg/+) were also statistically comparable to that in the wild-type (+/+) zygotes (*p* > 0.05, one way-ANOVA; Fig. 3a). The rate of multiallelic mosaic mutation tended to be lower in maCas9-carrying transgene-free zygotes when compared with that from Tg/+ zygotes and wild-type (+/+) zygotes, whereas the rate of non-mosaic mutation (50% indel mutation) tended to be higher. We also assessed possible off-target mutations caused by the injection of R2-gRNA into maCas9-carrying zygotes. When three candidate genes for the *Ramp2* locus were examined using maCas9-carrying zygote-derived blastocyst samples, there was no off-target mutation in these listed genes (Table S8). Thus, the activity of maCas9-based genome editing appears to be comparable to that of the zygote microinjection–based genome editing system used widely at present.

### maCas9 enables the mutation of nine loci simultaneously in wild-type zygotes

**Figure 4:**
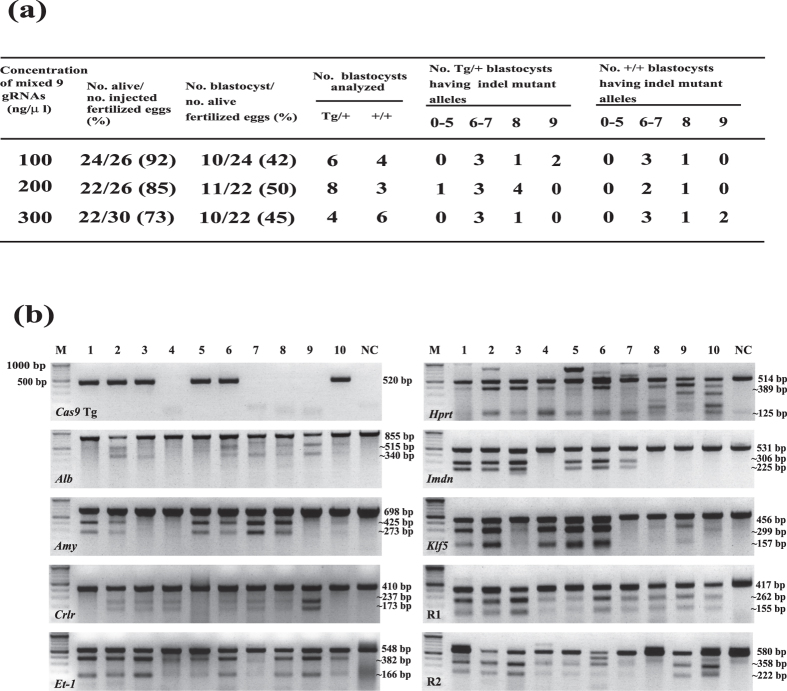
Demonstration of a maCas9-based multiple indel mutations induced simultaneously in Cas9 zygotes in the absence of the Cas9 transgene. (**a**) The generation rate of multiple indel mutant alleles in blastocysts derived from Cas9 zygotes (Cas9/+ female × +/+ male) injected with nine different gRNAs. Mixed 9 gRNAs; *Alb*-, *Amy*-, *Crlr*-, *Et-1*-, *Hprt*-, *Imdn*-, *Klf5*-, R1- and R2-gRNA were used (Figs 2c and 6b and Fig. S7). Pronuclear injections were performed with Cas9 zygotes (Cas9/+ female × +/+ male). Each indel mutant allele was identified by T7EI-based single blastocyst assay. (**b**) Gel image of Cas9 Tg genotyping and T7EI assay of 10 blastocysts created after pronuclear injection of mixed 9 gRNAs (100 ng/μl) shown in (**a**) as an example.

We previously found that maCas9 present in the Cas9 mouse-derived zygotes has a high degree of genome editing activity (Figs 2 and 3, Figs S5 and S6). To examine the number of mutations maCas9 could induce simultaneously in non-transgenic progeny, we injected nine different gRNAs into the pronucleus of zygotes derived from Tg/+ and +/+ IVF (Fig. 4). As controls, synthesized Cas9 mRNA and the nine gRNAs were co-injected into zygotes prepared from *+*/+ and +/+ IVF (Table S3). Cultured blastocysts were then subjected to Cas9 transgene genotyping and a T7EI-based cleavage assay. The results showed that blastocysts in the experimental group exhibited simultaneous mutations at multiple loci (7.3 alleles in average), which is roughly equivalent to the frequency obtained in the control group (Table S3). Notably, all nine targeted loci were mutated in two of the blastocysts examined, as well as two of the blastocysts tested in Cas9 transgenic zygotes. Furthermore, zygotes (42–50%) in the experimental group successfully developed *in vitro* into blastocysts; this was only observed in 5–19% of controls (Table S3). Thus, Cas9 transgene-free maCas9-containing zygotes may be useful for the simultaneous production of genetically modified mice with mutations at multiple loci.

### *In vitro* genome editing of α-1,3-galactosyltransferase (*Ggta1*) gene in Cas9/+ fibroblasts is achieved by transfection with gRNA alone

**Figure 5:**
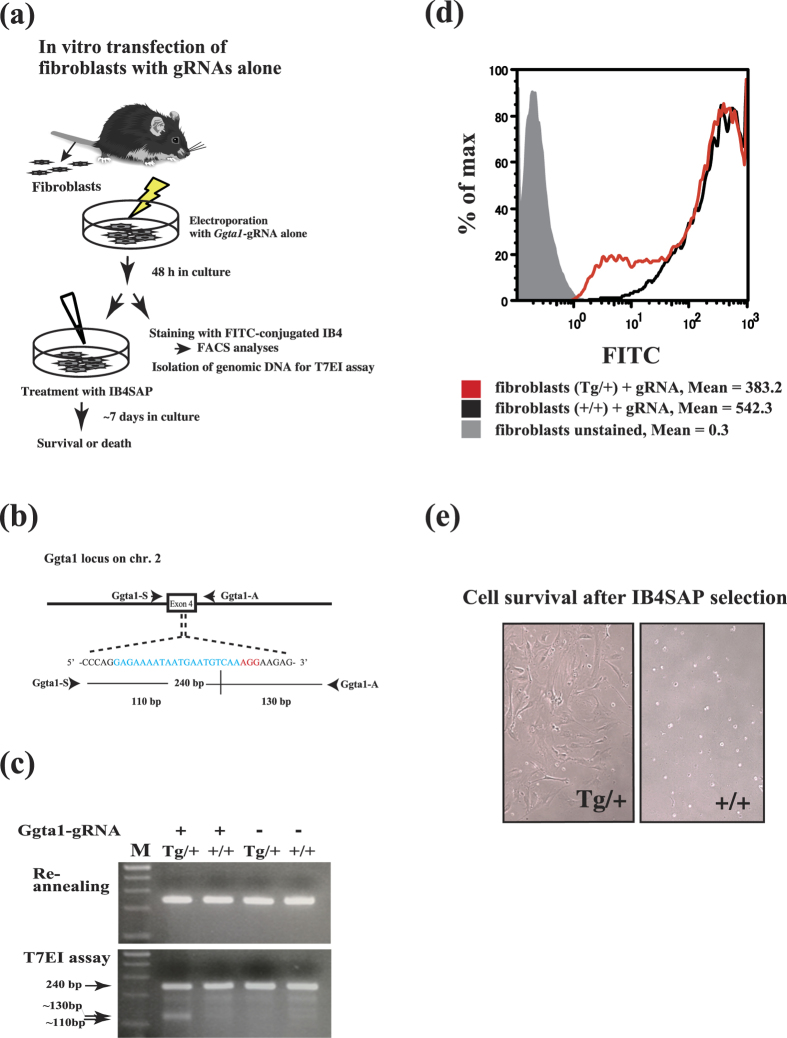
Induction of indel mutations in primary Cas9 Tg fibroblasts after electroporation-based transfection with α-1,3- galactosyltransferase (Ggta1)-gRNA alone. (**a**) Illustration of the procedures for induction of gRNA-mediated indel mutations in Cas9/+ cells *in vitro*. A.I drew the original mouse by hand (Fig. 2a), and T. Sa modified it by his permission. (**b**) Ggta1-gRNA targeting exon 4 of the murine Ggta1 gene. The Ggta1-gRNA-coding sequence is shown in blue. The PAM sequence is shown in red. PCR with Ggta1-S/Ggta1-A primers generates a 240-bp amplicon, from which two fragments (110- and 130-bp in size) are expected to be released through T7EI cleavage. (**c**) The T7EI assay showing the two expected bands only in the Cas9/+ fibroblasts transfected with *Ggta1*-gRNA alone. (**d**) Flow cytometric analysis of cell-surface α-Gal epitopes in the Cas9/+ fibroblasts transfected with *Ggta1*-gRNA alone. (**e**) A cell survival assay of *Ggta1*-gRNA transfected Cas9/+ and +/+ fibroblasts using saporin-conjugated BS-I-B_4_ lectin (IB4SAP).

Next, to verify the utility of Cas9 mice for genome engineering *in vitro*, we destroyed the endogenous *Ggta1* loci, whose product synthesizes the cell-surface carbohydrate α-Gal epitope (Fig. 5a,b). Primary fibroblasts derived from Cas9 mice (Tg/+) and their non-Tg offspring were then transiently transfected with *Ggta1* gRNA alone. T7EI assays revealed that only Tg fibroblasts harbored indel mutations within the *Ggta1* gene (Fig. 5c). Moreover, fluorescence activated cell sorting analysis demonstrated that only Tg fibroblasts exhibited a reduced expression of α-Gal epitope (~30% reduction when compared to non-Tg cells), which is specifically recognized by the FITC-labeled isolectin, BS-I-B_4_ (IB4) (Fig. 5d). Treatment of these transfected fibroblasts with toxic saporin-conjugated IB4 (IB4SAP) resulted in the death of *Ggta1* +/+ and KO/+ cells. In contrast, biallelic *Ggta1* KO cells survived after IB4SAP treatment, as they are unable to synthesize α-Gal epitope. Since only 20 surviving colonies were found from the 5 × 10^5^ cell in the treated culture, the KO efficiency was estimated to be ~0.004% (Fig. 5e). Thus, primary cells prepared from the Cas9 Tg line have potential to undergo CRISPR/Cas9-mediated genome editing after the administration of gRNA alone.

### *In vivo* genome editing of the albumin gene in Cas9/+ hepatocytes is achieved through hydrodynamic gRNA delivery

**Figure 6:**
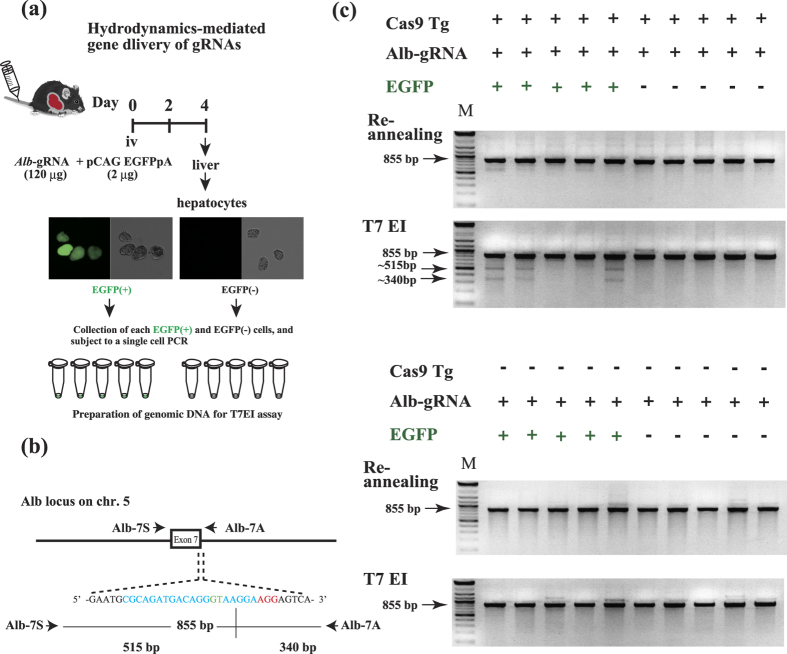
Indel mutation of the albumin (Alb) gene in Cas9/+ hepatocytes after transfection with Alb-gRNA alone by hydrodynamic-based gene delivery. (**a**) Schematic of the experimental methods. A.I drew the original mouse by hand (Fig. 2a), and T.Sa modified it by his permission. (**b**) gRNA targeted the vicinity of exon 7 in the murine Alb gene. The Alb-gRNA-coding sequence is shown in blue. The donor splice site, GT, is shown in green. The PAM sequence is shown in red. PCR using an Alb-7S/Alb-7A primer set generates 855-bp PCR products, from which two fragments (515- and 340-bp in size) are expected to be released by T7EI cleavage. (**c**) Upper panels: gel images of indel mutation in a single *Cas9* Tg hepatocyte co-transfected with *Alb*-gRNAs and pCAG-EGFPpA and analyzed by a T7EI assay. Lower panels: gel images of indel mutation in single wild-type hepatocytes co-transfected with *Alb*-gRNAs and pCAG-EGFPpA and analyzed by a T7EI assay. Experiments were repeated 3 times.

Finally, to demonstrate that Cas9 mice permit *in vivo* genome engineering upon gRNA administration, hydrodynamic gRNA delivery was employed to generate gene KO hepatocytes (Fig. 6a,b). For this, hepatocytes were isolated from the Tg or non-Tg mice following injection of albumin (*Alb*) gRNA and pCAG-EGFPpA into the tail vein. This method yielded EGFP(+) hepatocytes at a ratio of 0.1% (1/1000 cells). A total of 30 EGFP-positive and 10 EGFP-negative cells were isolated from Tg hepatocytes. Similarly, 10 EGFP-positive and 10 EGFP-negative cells were isolated from non-Tg mice. T7EI assays using genomic DNA from a single hepatocyte revealed that only EGFP-positive Tg hepatocytes harbored indel mutations in the *Alb1* gene at an average rate of 50% (15/30) (Fig. 6c), indicating that the percentage of mutated hepatocytes was about 0.05% (1/2000 cells). No indel mutations were observed in EGFP-negative Tg, EGFP-positive non-Tg, or EGFP-negative non-Tg hepatocytes. Altogether, these findings show that *in vivo* gDNA administration results in induction of indel mutations in the endogenous target gene of Cas9 mice.

## Discussion

The creation of Cas9-expressing cells or animals is likely to simplify the procedure to generate genetically modified animals with the CRISPR/Cas9 system, as only gRNAs would need to be administered to induce mutations at target loci. Since Cas9 is derived from bacteria and archaea, it is highly conceivable that its continuous expression in mammals would hinder viability; however, our data indicated that mice with multiple *Cas9* transgene copies were phenotypically normal and fertile. This finding is consistent with that of Platt *et al*.^20^, who produced a conditional Cas9-expressing line with one copy of the transgene that had been knocked-in in the *Rosa26* locus on chromosome 6. In addition, their findings also demonstrate the utility of Cas9-expressing mice for the study of lung carcinogenesis only by the *in vivo* viral administration of *Kras*, *Trp53*, and *Stk11*-gRNAs. Moreover, we also show that systemic Cas9 expression conferred genome editing activity both *in vivo* and *in vitro* upon administration of gRNA alone to primary fibroblasts by electroporation (Fig. 5) and to the liver by hydrodynamic delivery (Fig. 6). Thus, these results suggest that Cas9 mice serve as a useful tool to generate genetically modified animals that are considered as disease models or to prepare gene-engineered primary cells known to be difficult to establish as continuous cell lines. Once these model mice are established, they can afford repeatable genome engineering owing to sustained systemic expression of active Cas9. For example, it is often difficult to insert both Cas9 and gRNA expression sequences into a single viral vector due to size limitations^23^. In this regard, Cas9 mice would be useful for researchers who want to perform genome editing by administering virally packaged gRNAs or DNA fragments/oligonucleotides or with multiple tissue-specific KO genotypes on an individual level^24^,^25^,^26^.

Most importantly, Cas9 mice will provide an opportunity to edit multiple target loci simultaneously by injecting zygotes with gRNAs alone (Figs 2, 3, 4, Figs S5 and S6). Our study demonstrated that Cas9 transgene-free maCas9-containing zygotes exhibit a high degree of genome editing sufficient to create genetically modified mice with multiple KO genotypes. Furthermore, these zygotes appeared to develop normally after the pronuclear injection of gRNA alone, and demonstrated an efficiency comparable to those with *Cas9* mRNA and gRNA (or gRNA expression vector) co-injections^11^,^12^,^14^,^17^,^18^,^19^. The maternal factors that accumulate in oocytes during oogenesis are functional from the oocyte to the 2-cell stage, and are degraded thereafter^22^. Thus, the maCas9 activity present in derived zygotes should function specifically at the zygote stage. We observed that the genome-editing activity of zygotes derived from IVF of Tg/+ oocytes and +/+ sperm is higher than that from IVF of +/+ oocytes and Tg/+ sperm (Fig. 2b). Since this form of genome editing is transient and occurs in the absence of the *Cas9* transgene, we have termed it “non-inheritable maternal Cas9-based genome editing (maCas9GED).” This system has several advantages over the pre-existing method based on zygote injection. For example, since maCas9 activity is considered higher than that of *in vitro* synthesized *Cas9* mRNA, researchers should be able to generate genetically modified animals with greater efficiency in the absence of a continued supply of Cas9 materials (Figs 2 and 3, Figs S5, and S6). Moreover, the transience of maCas9 may also decrease the generation of mosaic cells in F0 animals, since the rate of non-mosaic mutation (monoallelic 50% indel mutation) tended to be higher in maCas9-carrying transgene-free zygotes when compared with that from Tg/+ zygotes and wild-type (+/+) zygotes (Fig. 3a). If non-Tg zygotes derived from pairs of Tg/+ females and Tg/+ or +/+ males are available, it is possible to produce *Cas9* transgene-free gene-modified mice (Figs 2, 3, 4 and Fig. S6). This is especially advantageous for people who wish to explore how maCas9 affects the mode of genome editing during pre-implantation embryogenesis. Moreover, maCas9GED would also facilitate the production of genetically modified large animals—such as pigs and bovines—which could be marketed to consumers as “transgene-free animals.”

Finally, one of the primary aims of this study was to determine if Cas9 zygotes could undergo mutation at multiple target loci simultaneously. Currently, the production of CRISPR/Cas9 genetically modified animals requires the co-microinjection of *Cas9* DNA (or mRNA) and a gRNA expression vector (or gRNA) into zygotes^8^,^9^,^10^,^11^,^12^,^14^,^15^,^17^,^18^,^19^. In a preliminary test, we found that *Cas9* DNA (or mRNA) can restrict the capacity of a zygote to uptake multiple gRNAs because of limitations on the injection volume; however, the use of Cas9 zygotes would likely extend this capacity. In fact, at least nine target loci were mutated simultaneously in maCas9 zygotes even in the absence of the *Cas9* transgene (Fig. 4 and Table S3). Thus, the maCas9GED system would be beneficial for researchers aiming to produce *Cas9* transgene-free multiplexed KO or knock-in animals as multifactorial human disease model animals, which are considered to be difficult to generate with pre-existing methods. Thus, we plan to focus our efforts on exploring the conditions that will allow efficient gene editing in zygotes injected with several gRNA constructs or homologous fragments^27^,^28^,^29^.

## Methods

### Ethics statement

All mouse experiments were conducted in according with institutional guidelines and were approved by the Animal care and Use committee of Shinshu University (Approval Number # 250035).

### DNA construction and gRNA synthesis

Two transgenes, pCAG-NFCas9 and pCAG-FCas9, were first constructed to generate *Cas9* Tg mice. The sequence encoding hCas9 was PCR-amplified using phCas9 (Addgene; Cambridge, MA, USA) as a template with primers Cas9ATG-S and Cas9Cla-A. Then, the SV40-derived nuclear location signal (NLS) and FLAG sequences or FLAG sequence alone were placed immediately after the ATG codon of the *hCas9* sequence by PCR. The resulting constructs were termed “NFCas9” (NLS + FLAG) and “FCas9” (FLAG only), respectively. These expression units were then sub-cloned into pCAGGS^30^, which had been modified to possess two NotI sites at the 5′ end of the cytomegalovirus enhancer and the 3′ end of poly(A) sites. The fidelity of these constructed plasmids was then confirmed by sequencing. The Cas9-encoding transgene was then excised from the vector digestion with NotI prior to microinjection. The gRNA sequences and PCR primers used in this study are summarized in Tables S4, S5, and S6, respectively.

To generate a series of pregenomic RNAs (pgRNAs)—including pAlb-gRNA, pAmy-gRNA, pCrlr-gRNA, pEt-1-gRNA, pHprt-gRNA, pImdn-gRNA, pKlf5-gRNA, pR1-gRNA and pR2-gRNA—the 83-bp sequence spanning from the gRNA scaffold to the TTTTTT site was PCR-amplified using pgRNA_GFP-T1 (#41819; Addgene) as a template and then sub-cloned into pBluescript II (Agilent Technologies Japan, Ltd.; Tokyo, Japan). The resulting plasmid, pBS-T7-gRNA, was then used as a template to add a series of target sequences (Table S4) 5’ of the gRNA scaffold sequence in pBS-T7-gRNA by inverse PCR. The constructed plasmids were confirmed by sequencing. A series of gRNAs were prepared from an EcoRI-linearized plasmid using the Ambion MEGAshortscript T7 kit (Life Technologies Japan, Ltd.). *Cas9* mRNAs were prepared from SapI-linearized pBS-NFCas9 using the Ambion mMESSAGE mMACHINE T3 kit (Life Technologies Japan, Ltd.)^31^.

### Generation and characterization of Cas9 mice

BDF1, C57BL6/J-Jcl (hereafter termed B6), and ICR mice were purchased from CLEA Japan Inc. (Tokyo, Japan). Zygotes were collected from BDF1 females using a standard *in vitro* fertilization (IVF) protocol with a B6 male^32^. Tg mice was generated as previously described with pronuclear DNA injections (2 ng/μL)^33^. Microinjected zygotes were transferred into the oviducts of pseudopregnant ICR surrogate females. Identification of founder *Cas9* Tg mice and their progenies was performed by PCR using the primer set CAG1620-S/CAS9215-A.

qRT-PCR was performed to determine the transgene copy-number in Tg mice using NFCas9 and FCas9 DNA copy standard curves (Fig. S2b) and Cas9E-S/Cas9Cla-A primers. Cas9 standard curves were prepared from eight different PCRs using a DNA solution containing 100 ng of genomic DNA and serial dilutions of pCAG-NFCas9 or pCAG-FCas9 (1, 2, 4, 8, 16, 32, 64, and 128 haploid copies per 100 ng of genomic DNA) as templates. *Cas9* mRNA expression levels in Tg mice were measured by qRT-PCR using Cas9E-S and Cas9cla-A primers and NFCas9 RNA copy standard curves, as well as by CAG1620-S/Cas9215-A and Ramp3-S/Ramp3-A qRT-PCRs and western blotting with anti-FLAG; F1804, Sigma-Aldrich; St. Louis, MO, USA). mRNA copy number standard templates were prepared in a solution containing total RNA (50 ng) isolated from a wild-type 13.5-dpc embryo and serially diluted (10, 10^2^, 10^3^, 10^4^, and 10^5^ copies) *in vitro*-synthesized *Cas9* RNA (sequence length: 4146 nucleotides; 0.18 pg corresponding to 10^5^ copies). Next, cDNAs were reverse-transcribed using both random and oligo dT primers. As a result, the standard curve was obtained from five different PCRs using 2 μL of cDNA template (equivalent to 50 ng of total RNA) (Fig. S2c). The body weights of Tg mice and their non-Tg littermates were measured every week from the age of 4 to 10 weeks old. BUN, Cr, AST, and ALT serum levels were assessed in 10-week-old mice by SRL, Inc. (Tokyo, Japan). Histological analyses were performed using a standard hematoxylin and eosin staining procedure. Briefly, tissues were fixed overnight in cold 4% paraformaldehyde/phosphate-buffered saline, embedded in paraffin, and cut into 5–6-μm-thick sections. The chromosomal localization of *Cas9* transgenes in the NFCas9-2 Tg line was determined by splinkerette PCR^34^, using the EcoRV splinkerette oligonucleotides (sp-EV Top and sp-Bottom). To confirm whether the results obtained from splinkerette PCR analysis were accurate, PCRs of the genomic DNA was performed with primers including Gtpbp10-S, Sp#2, Cas9E-S, CAGGS-A, and Gtpbp10-A.

### RNA-microinjection into Cas9 zygotes and indel mutation analysis

Zygotes were prepared from various female–male pairs using standard IVF techniques (Fig. 2a,b). Microinjection of gRNA(s) into the pronuclei/cytoplasm of zygotes was performed as previously described^31^. Microinjected zygotes were cultivated *in vitro* in a potassium-supplemented simplex-optimized (KSOM) medium until the blastocyst stage^31^ or immediately transferred to the oviducts of pseudopregnant ICR females.

The developing blastocysts were subjected to a T7EI assay based on a single mouse blastocyst^31^ using T7 endonuclease I (T7EI, New England Biolabs Japan Inc.; Tokyo, Japan), and *Cas9* Tg genotyping. For example, Ramp1-2S/Ramp1-2A for identification of mutations in the *Ramp1* locus, and Ramp2-5S/Ramp2-6A for identification of mutations in the *Ramp2* locus. In some cases, the resulting PCR products were subjected to direct sequencing using BigDye Terminator v.3.1 and ABI Genetic Analyzer 3130 (Applied Biosystems, Life Technologies Japan, Ltd., Tokyo, Japan). In addition, genomic fragments containing top three off-target candidate genes with respect to the *Ramp2* on-target site were PCR-amplified and sequenced using the above system. PCR primers used for *Cas9* genotyping were CAG1620-S and CAS9215-A.

Fetuses were collected from pregnant ICR females 13.5–15.5 days after transplantation of gRNA-microinjected zygotes. The crude DNA solutions prepared from the tails or hands of mice were used as PCR templates, and also subjected to a T7EI assay and genotyping as described above. In some cases, the resulting PCR products were subjected to direct sequencing. PCR products for top three off-target candidate genes for *Ramp1* or *Ramp 2* genes were also subjected to direct sequencing, as described above.

*Ramp1* and *Ramp2* mRNA expression in the dissected fetuses were assessed by SYBR green-based qRT-PCR using a total RNAs and a cDNA synthesis kit (TaKaRa, Bio, Inc.; Shiga, Japan). The following primer sets were used: Ramp1-SF/Ramp1-SR for *Ramp1* mRNA, Ramp2-SF/Ramp2-SR for *Ramp2* mRNA, and HPRT-SF/HPRT-SR for hypoxanthine phosphoribosyltransferase (*Hprt*) mRNA as a reference gene.

### *Ggta1*-gRNA electroporation into Cas9/+ fibroblasts and *Ggta1* mutational analysis

The methodology for this experiment is illustrated in Fig. 5a, which is based on the methods used in Sato *et al*.^35^ with some modifications. Briefly, tail snips (1.5 cm) from Tg mice and their non-Tg littermates were minced using sterile scissors, placed on a tissue-culture dish containing high-glucose Dulbecco’s modified Eagle’s essential medium supplemented 10% fetal bovine serum, and then cultured for 5 days. Cells that migrated from the tail pieces were passaged 2–3 times. Approximately 5 × 10^6^ cells were suspended in phosphate-buffered saline without Ca^2+^ and Mg^2+^ (pH 7.4), supplemented with 100 μg/mL *Ggta1*-gRNA, and electroporated with Gene-Pulser II (Bio-Rad; Hercules, CA, USA) at 25 puF and 1.35 kV prior to seeding in a 100-mm tissue culture dish. Two days after transfection, cells were harvested and subjected to flow cytometric analysis to examine cell-surface α-Gal epitope expression. Alternatively, some cells were subjected to a T7EI-based assay using Ggta1-S/Ggta1-A primers that correspond to the exon 4 target region of the *Ggta1* gene (Fig. 5b). Fluorescein isothiocyanate-conjugated BS-IB_4_ isolectin (I21411; Life Technologies Japan, Ltd.) capable of binding specifically to α-Gal epitope was used to discriminate between α-Gal epitope-positive and -negative cells. Furthermore, survival assays with saporin (SAP)-conjugated BS-I-B_4_ (#IT-10; Advanced Targeting Systems Inc.; San Diego, CA, USA) were performed to obtain fibroblasts with a biallelic *Ggta1* KO genotype.

### Hydrodynamic gRNAs delivery into Cas9*/+* mice and indel mutational analysis in hepatocytes

The procedure for hydrodynamic gRNA delivery is shown in Fig. 6a. Hydrodynamic gRNA delivery was carried out using the TransIT-QR Hydrodynamic Delivery Solution kit (Mirus Bio LLC; Madison, WI, USA) according to the manufacturer’s protocol. Albumin (*Alb*) gRNA (120 μg) and pCAG-EGFPpA (4 μg) were also injected into the tail vein. pCAG-EGFPpA^36^ was used as a reference gene to monitor the co-introduction of *Alb* gRNA.

Hepatocytes were prepared as previously described^37^. Single hepatocytes were collected using a standard microinjection system (Narishige Group, Ltd.; Tokyo, Japan) attached to an Olympus IX70 inverted fluorescence microscope with a U-MWIBA2 filter set (Olympus; Tokyo, Japan). A single hepatocyte was subjected to analyses as previously described for a single mouse blastocyst assay^31^. Briefly, three rounds of PCR were performed to amplify an 855-bp region of the murine *Alb* gene (Fig. S7). For the first round of PCR, a 23-μL reaction mixture containing 1× Tks Gflex buffer (TaKaRa Bio, Inc.), 0.4 μM each primer (Alb-7S and Alb-7A), and 1.25 U of Tks Gflex DNA polymerase (TaKaRa Bio, Inc.) was mixed with 2 μL of crude DNA^38^, which was prepared from single hepatocytes. PCRs were performed by denaturation at 94 °C for 1 min, followed by 35 cycles of 98 °C for 5 s, 68 °C for 20 s, and 68 °C for 5 min. For the second and third rounds of PCR, 2 μL of the PCR product from the first and second PCR rounds was added to the 23-μL reaction mixture, as described above. The PCR conditions were the same as those used for the first PCR throughout the second and third rounds of PCR.

## Additional Information

**How to cite this article**: Sakurai, T. *et al*. A non-inheritable maternal Cas9-based multiple-gene editing system in mice. *Sci. Rep*. **6**, 20011; doi: 10.1038/srep20011 (2016).

## Supplementary Material

Supplementary Information
