# Supplementary material for: A non-inheritable maternal Cas9-based multiple-gene editing system in mice
Source: Sci Rep. 2016 Jan 28;6:20011. doi: 10.1038/srep20011 (PMC4730228; doi:10.1038/srep20011)
Supplement: Supplementary Information [file srep20011-s1.pdf]

Scientific Reports

Supplementary information

A non-inheritable maternal Cas9-based multiple-gene editing system in mice

Takayuki Sakurai[1*], Akiko Kamiyoshi[1], Hisaka Kawate[1], Chie Mori[1], Satoshi Watanabe[2], Megumu Tanaka[1], Ryuichi Uetake[1], Masahiro Sato[3], Takayuki Shindo[1]

[1]Department of Cardiovascular Research, Graduate school of Medicine, Shinshu University, 3-1-1 Asahi, Matsumoto, Nagano 390-8621, Japan

[2]Animal Genome Research Unit, Division of Animal Science, National Institute of Agrobiological Sciences, 2-1-2 Kannondai, Tsukuba, Ibaraki 305-8602, Japan

[3]Section of Gene Expression Regulation, Frontier Science Research Center, Kagoshima University, 8-35-1 Sakuragaoka, Kagoshima, Kagoshima 890-8544, Japan

***Correspondence: tsakurai@shinshu-u.ac.jp**

Supplementary figures

Supplementary tables

Supplementary figures

(a)

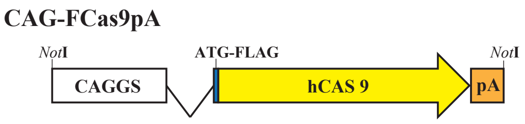

(b)

Generation of Cas9 transgenic mice

Construct	No. zygotes injected	No. zygotes transferd	No. newborn mice	No. pups	No. transgenic mice	Integration efficiency(%)	Overall efficiency(%)
NFCas9	56	45	16	13	4	4/13 (31)	4/56 (7)
FCas9	83	75	17	13	5	5/13 (38)	5/83 (6)

(c) NFCas9

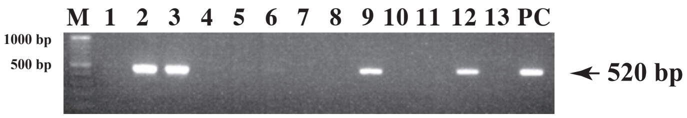

(d) FCas9

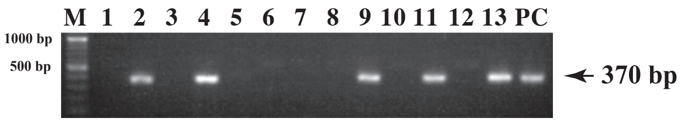

Figure S1.

Figure S1. Generation of a transgenic (Tg) mouse line with ubiquitous expression of humanized Cas9. (a) Schematic of the FCas9 transgene used for generating Cas9 Tg mice. (b) Summary of the production efficiency for *Cas9* Tg mice after pronuclear introduction with two transgene constructs, NFCas9 and FCas9. (c-d) Tg founder mice were identified by PCRs with genomic DNA isolated from pups developed from microinjected zygotes. PCR products were electrophoresed using 1% agarose gel/TAE buffer. M, lambda HindIII + 100-bp ladder; PC, positive control (pCAG-NFCas9 or pCAG-FCas9 plasmid). (c) Among Tg mice produced carrying NFCas9, four (Nos. 2, 3, 9, and 12) of 13 pups were identified as Tg. (d) Among Tg mice carrying FCas9, five (Nos. 2, 4, 9, 11, and 13) of 13 pups were identified as Tg.

(a)

Construct	Name of founderTg	Estimated copy no.[1]	Sex.	F1 offspring[2] ♀(Tg/+: +/+)	♂(Tg/+: +/+)	Transegene transmission to F1 offspring	Transegene expression[3]
NFCas9	2	9	♂	♀(2:27)	♂(6: 16)	YES	+++ [4]
	3	>128	♀	♀(0:10)	♂(0: 8)	NO	ND
	9	17	♀	♀(2:6)	♂(1: 6)	YES	+
	12	1	♂	♀(0:9)	♂(0: 6)	NO	ND
FCas9	2	98	♂	♀(3:10)	♂(2: 5)	YES	+
	4	40	♂	♀(2:2)	♂(2: 5)	YES	+
	9	117	♂	♀(5:9)	♂(9: 8)	YES	+
	11	>128	♀	♀(2:3)	♂(3: 4)	YES	-
	13	24	♀	♀(2:3)	♂(4: 5)	YES	++

1. Transgene copy numbers were estimated by Cas9 standard curves by real time qPCR (Figure S2B)
2. F1 offsprings were obtained by mating Tg mice with B6♀ or B6♂.
3. Transgene expression were determined by RT-PCR and WB (Figure S2D)
4. The copy number of the Cas9 mRNA expression per 50ng of total RNA was 3431± 95 (Figure 1B).

(b)

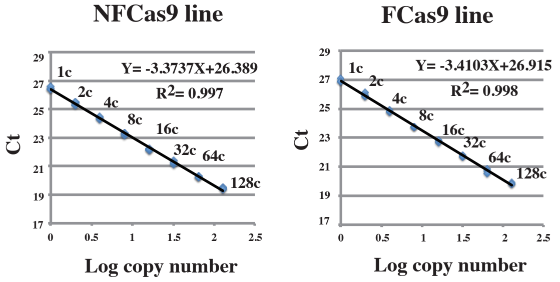

(c)

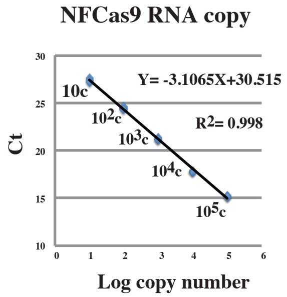

(d)

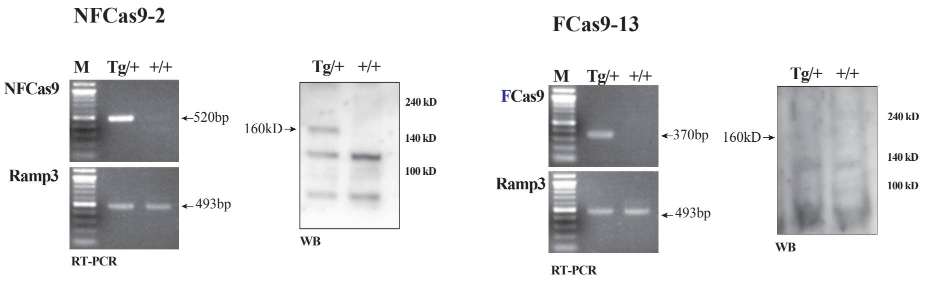

Figure S2.

Figure S2. Copy number, transmission, and expression of *Cas9* transgene in established *Cas9* Tg mouse lines. (a) Summary of the characteristics of the established *Cas9* Tg mouse lines. (b) The two standard curves for estimating the transgene copy number. Transgene copy numbers of 9 Tg mouse lines shown in (a) were determined based on these standards. The slopes of the two standard curves obtained were approximately -3.4, and their correlation coefficients (R^2) were >0.98, indicating nearly optimal reaction efficiencies. (c) The standard curve for estimating mRNA copy number. The slope of the standard curve obtained was approximately -3.1, and the correlation coefficient (R^2) was 0.998. *Cas9* mRNA copy numbers were estimated in the NFCas9-2 line was estimated based on this standard curve as shown in **Figure 1b**. (d) Stable *Cas9* expression in primary tail-derived fibroblasts of *Cas9* Tg mouse lines, NFCas9-2 and FCas9-13. Initially, *Cas9* mRNA expression in Tg/+ fibroblasts of seven Tg mouse lines shown in (a) was assessed by RT-PCR. Representative data from NFCas9-2 and FCas9-13 are shown. The *Ramp3* gene was used as positive control for RT-PCR. *Cas9* PCR products of expected 520- and 370-bp were detected only in NFCas9-2 and FCas9-13, respectively. Western blot analysis demonstrated the production of the expected 160-kDa proteins in both Tg lines. Among the seven lines tested, the NFCas9-2 line had the highest Cas9 expression, followed by the FCas9-13 line.

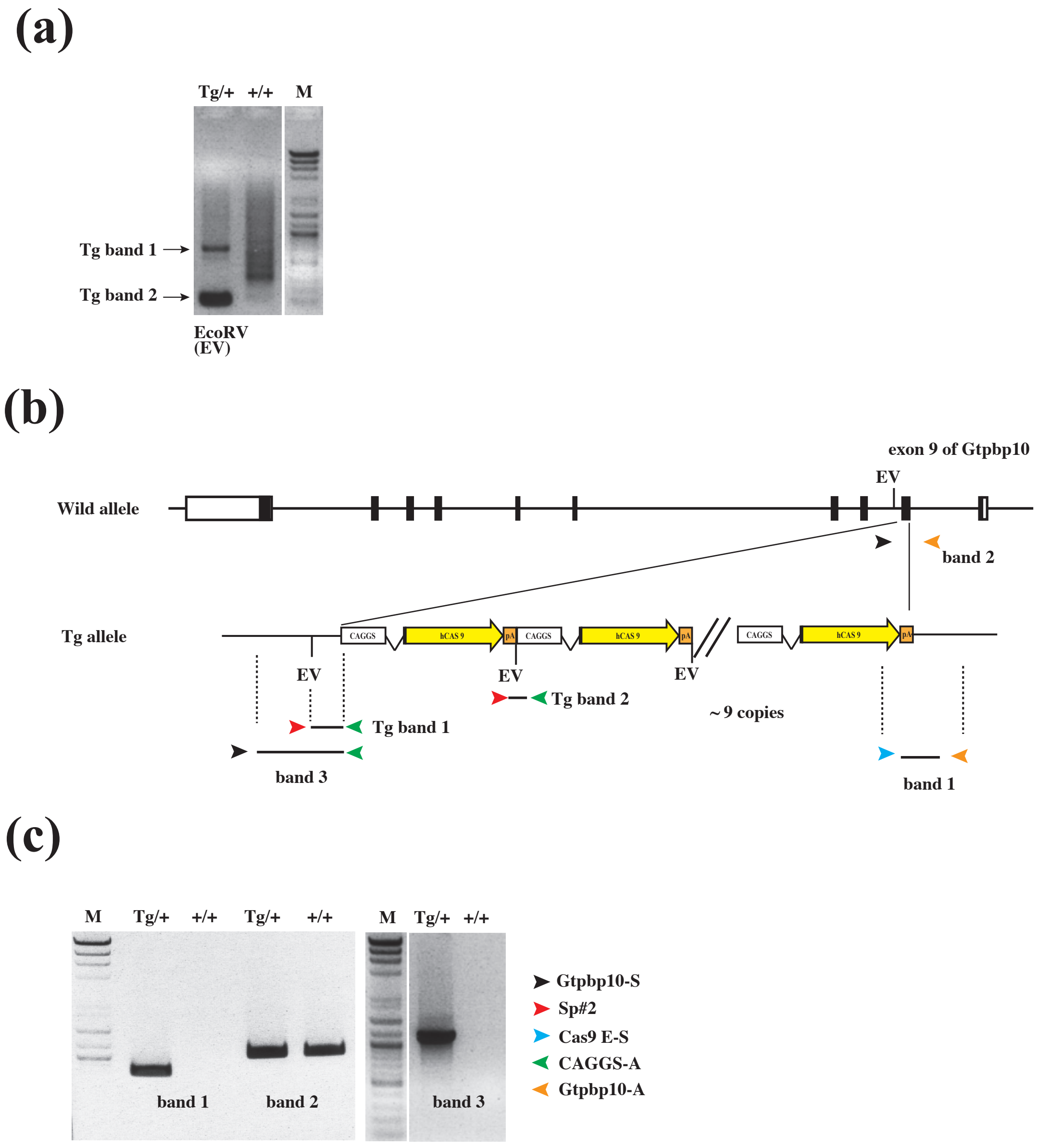
(a)
Tg/+
+/+
M
Tg band 1
Tg band 2
EcoRV
(EV)
(b)
exon 9 of Gtpbp10
EV
Wild allele
band 2
Tg allele
CAGGS
hCAS 9
pA
EV
Tg band 2
~ 9 copies
Tg band 1
band 3
band 1
(c)
M
Tg/+
+/+
band 1
band 2
band 3
Gtpbp10-S
Sp#2
Cas9 E-S
CAGGS-A
Gtpbp10-A

Figure S3.

Figure S3. The transgene integration site was mapped to exon 9 of the GTP-binding protein 10 (*Gtpbp10*) gene on chromosome 5 in the NFCas9-2 line carrying nine transgene copies (Figure S2a) by the Splinkerette (sp) PCR method. (a) The two Tg-specific spPCR products (Tg band 1 and Tg band 2) were detected by 1% agarose gel electrophoresis. The resulting two PCR products (Tg band 1 and Tg band 2) were subjected to direct sequencing and analyzed by BLAST. (b) The location and estimated integration pattern of the NF*Cas9* transgene into exon 9 of *Gtpbp10*. The positions of Tg band 1 and Tg band 2 are indicated in the map, but the overall structure of the integrated transgenes (such as a head-to-tail or tail-to-tail integration pattern) was not identified. (c) Data confirming the NF*Cas9* transgene integration map shown in (b) using the site-specific PCR primers. M, lambda HindIII + 100-bp ladder.

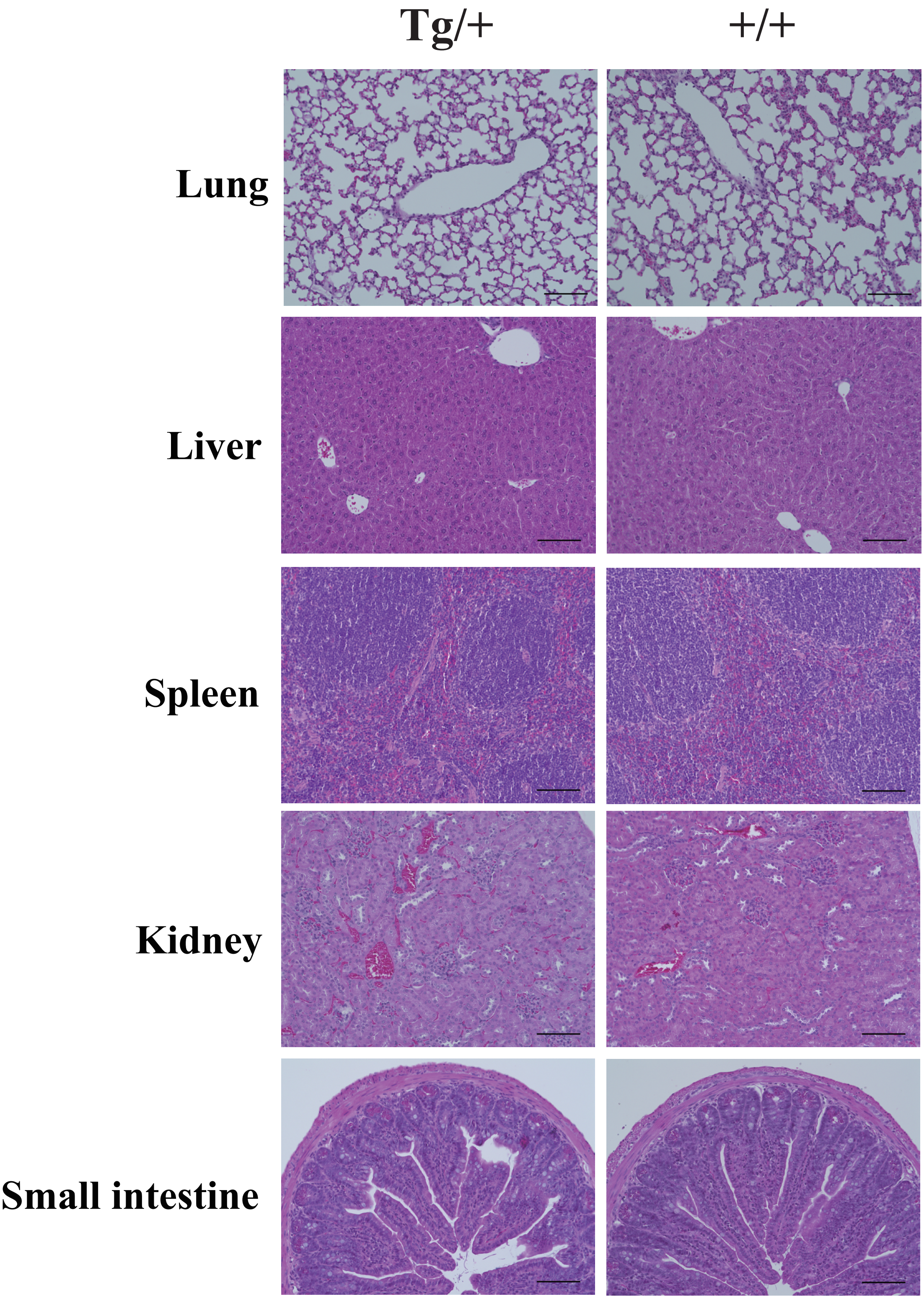

Figure S4.

Figure S4. Histological analyses in *Cas9* Tg mice. Organs were collected from 10-week-old Tg/+ and +/+ (each n = 3) NFCas9-2 mice. Sections were cut at 5-µm thickness and stained with hematoxylin and eosin. Representative images are shown. The heart, skeletal muscle, and testis showed the strongest *Cas9* expression at the mRNA level (**Figure 1d**). Scale bar = 100 µm.

(a)

gRNA (ng/μl)	No.alive zygotes/ no. injected zygotes (%)	No. blastocyst developed/ /no.injected zygotes (%)	No. blastocyst with mutated R1 /no. blastocyst tested (%)	No. blastocyst with mutated R2 /no. blastocyst tested (%)
R1(25)	19/20 (95)	7/19 (37)	7/7 (100)	0/7 (0)
R2(25)	10/13 (77)	6/10 (60)	0/6 (0)	5/6 (83)
R1+R2 (12+12)	16/21 (76)	9/16 (56)	9/9 (100)	9/9 (100)

(b)

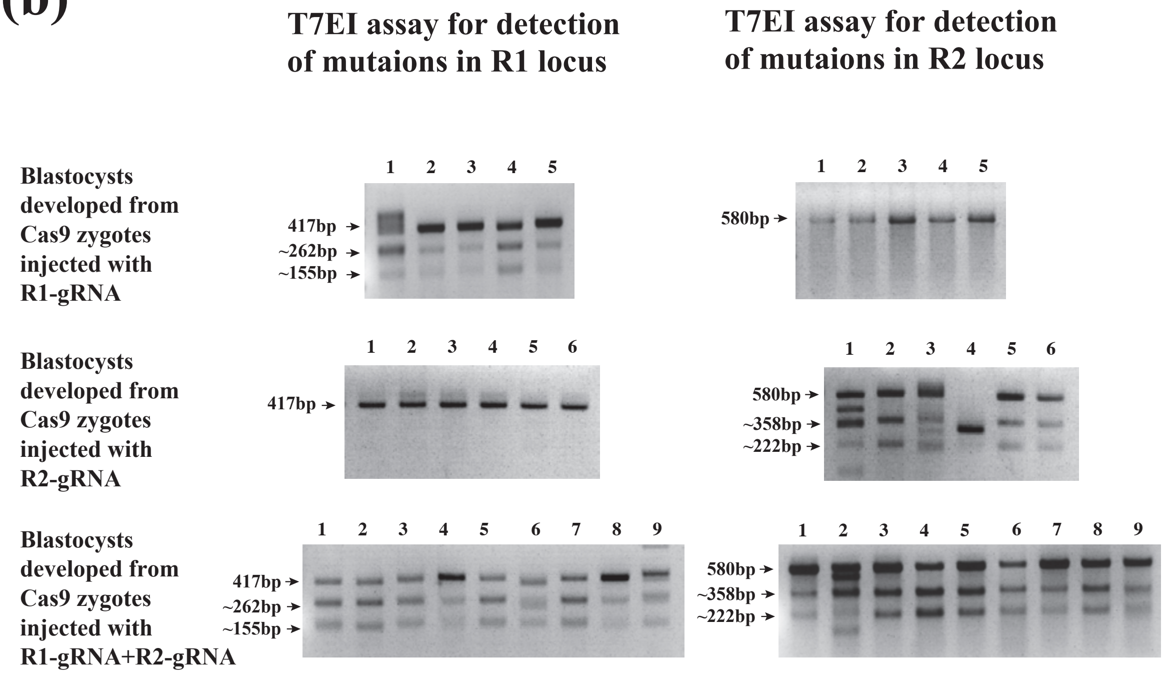

Figure S5.

Figure S5. gRNA-specific indel mutation in blastocysts derived from zygotes injected with R1-gRNA, R2-gRNA, or R1-gRNA + R2-gRNA. (a) Summary of the occurrence rate of gRNA-specific indel mutations when single blastocysts were examined by T7EI assay. (b) Gel images of the T7EI assay of each blastocyst shown in (a). The gRNA target sites in the murine *Ramp1* and *Ramp2* genes are schematically shown in **Figure S7** and **Figure 2c**, respectively.

(a)

Experiment no.	gRNA (ng/μl)	Alive/Injected Fertilized eggs (%)	Transferred (Recipient)	13.5-15.5dpc embryo/no. transferred (%)	Indel mutated/ analyzed embryos (%)	No. Tg embryos (indel hetero : homo)	No. + embryos (indel hetero : homo)
1	gR1+gR2 (12+12)	48/64 (75)	48 (3)	10/48 (21)	R1 8/10(80)<br>R2 8/10(80)	6 (6: 0)<br>6 (6: 0)	2 (2: 0)<br>2 (2: 0)
2	gR1 (25)	50/64 (78)	49 (2)	24/49 (49)	9/9 (100)	4 (4 : 0)	5 (4 : 1)
3	gR2 (25)	42/44 (95)	42 (2)	5/42 (12)	4/5 (80)	4 (3 : 1)	0 (0 : 0)

(b)

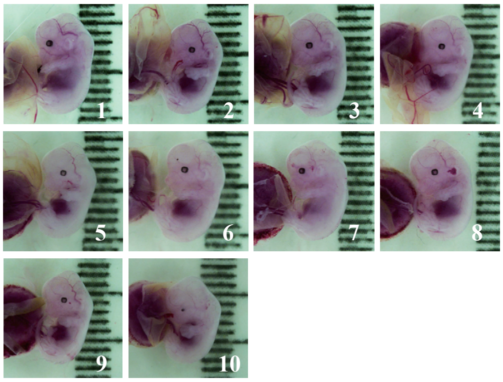

(c)

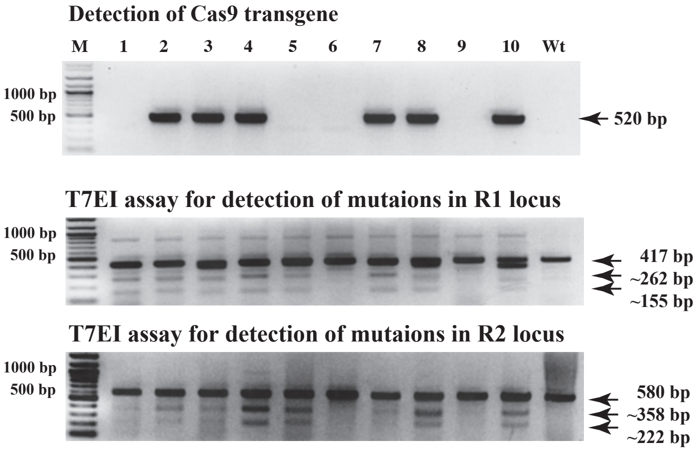

(d)

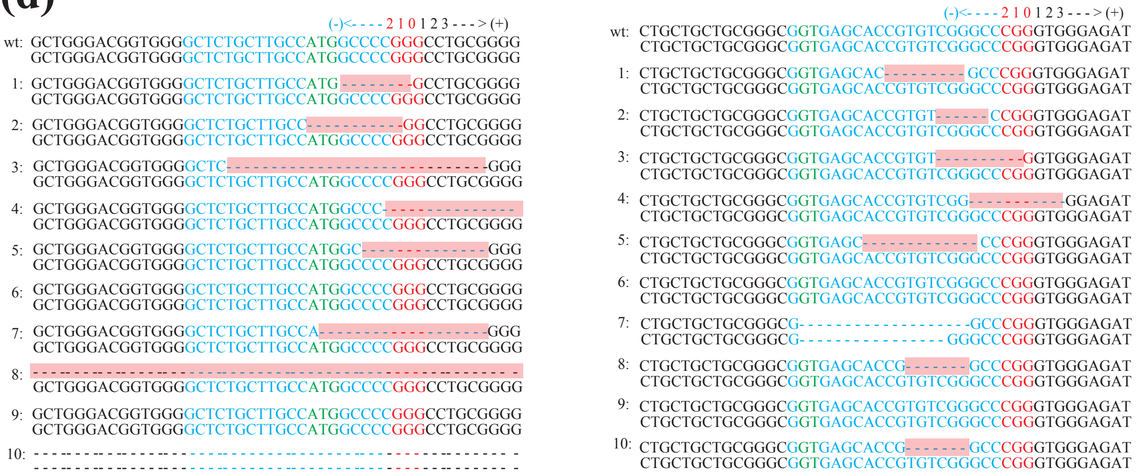

(e)

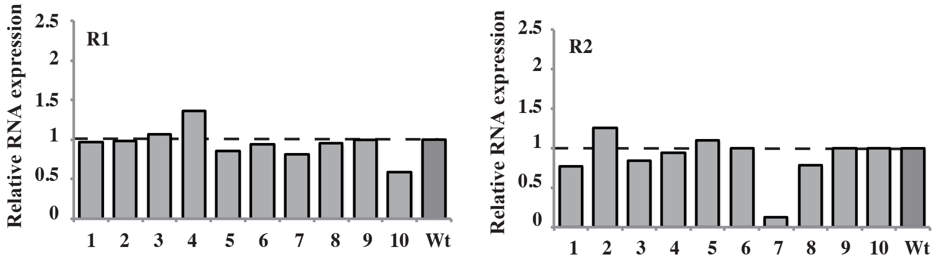

Figure S6.

Figure S6. Generation of R1 and R2 double-mutant mice using Cas9 zygotes in the absence of Cas9 transgene. (a) Occurrence of indel mutations in the offspring of Cas9/+ and +/+ mice after microinjection with *R1*-gRNA alone, *R2*-gRNA alone, or both gRNAs in zygotes. (b-e) Figures show the results from the 1st experiment summarized in (a). (b) Normal morphology of 10 fetuses at 13.5 dpc derived from fertilized eggs injected with both *R1*- and *R2*-gRNAs. (c) Gel images of *Cas9* genotyping (upper panel) and results from the T7EI assay to detect indel mutations in the *Ramp1* (middle panel) and *Ramp2* loci (lower panel) of the fetuses shown in (b). The location of gRNA targeting sites in the *Ramp1* gene is shown in **Figure S7**. (d) Sequencing of PCR products. (e) qPCR analyses. R1 and R2 values of Nos. 1–10 and wild-type (wt) fetuses were normalized to those of *Hprt*, and are shown as the relative expression ratio of that in wt controls (value of 1).

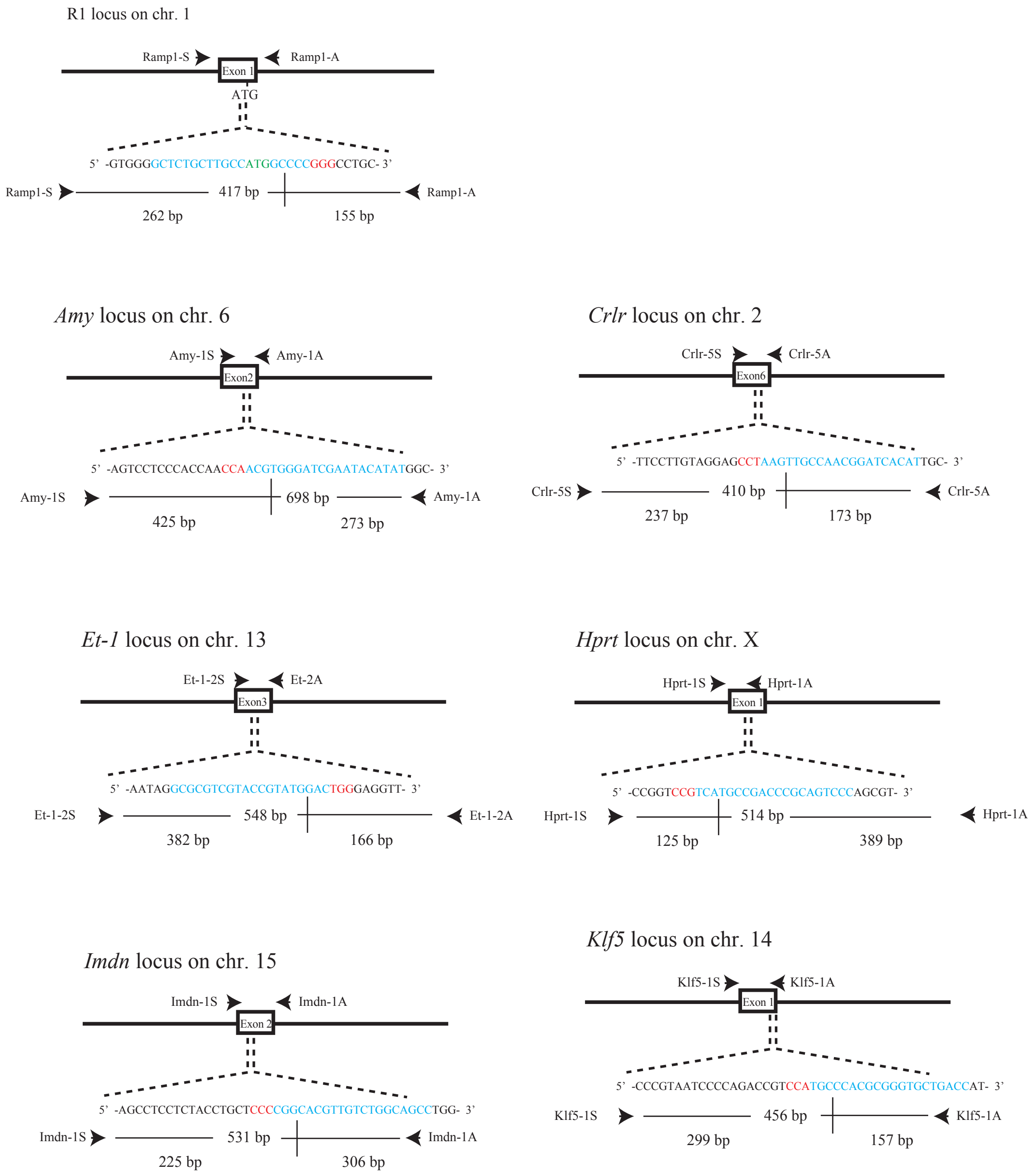

R1 locus on chr. 1
Ramp1-S
Ramp1-A
Exon 1
ATG
5' -GTGGGGCTCTGCTTGCCATGGCCCCGGGCCTGC- 3'
Ramp1-S
417 bp
Ramp1-A
262 bp
155 bp
Amy locus on chr. 6
Amy-1S
Amy-1A
Exon2
5' -AGTCCTCCCACCAACCAACGTGGGATCGAATACATATGGC- 3'
Amy-1S
698 bp
Amy-1A
425 bp
273 bp
Crlr locus on chr. 2
Crlr-5S
Crlr-5A
Exon6
5' -TTCCTTGTAGGAGCCTAAGTTGCCAACGGATCACATTGC- 3'
Crlr-5S
410 bp
Crlr-5A
237 bp
173 bp
Et-1 locus on chr. 13
Et-1-2S
Et-2A
Exon3
5' -AATAGGCGCGTCGTACCGTATGGACTGGGAGGTT- 3'
Et-1-2S
548 bp
Et-1-2A
382 bp
166 bp
Hprt locus on chr. X
Hprt-1S
Hprt-1A
Exon 1
5' -CCGGTCCGTCATGCCGACCCGCAGTCCCAGCGT- 3'
Hprt-1S
514 bp
Hprt-1A
125 bp
389 bp
Imdn locus on chr. 15
Imdn-1S
Imdn-1A
Exon 2
5' -AGCCTCCTCTACCTGCTCCCCGGCACGTTGTCTGGCAGCCTGG- 3'
Imdn-1S
531 bp
Imdn-1A
225 bp
306 bp
Klf5 locus on chr. 14
Klf5-1S
Klf5-1A
Exon 1
5' -CCCGTAATCCCCAGACCGTCCATGCCCACGCGGTGCTGACCAT- 3'
Klf5-1S
456 bp
Klf5-1A
299 bp
157 bp

Figure S7.

Figure S7. The information of six gRNAs for the chromosomal location and T7EI analyses.

Each of the six gRNA sequences for the murine genes *Amy*, *Crlr*, *Et-1*, *Hprt*, *Imdn*, and *Klf5* are shown. The gRNA-coding sequence is shown in blue. The PAM sequence is shown in red. For example, PCR using a Amy-1S/Amy-1A primer set generates 698-bp PCR products, from which two fragments (273- and 425-bp in size) are expected to be released by T7EI cleavage.

Supplementary tables

Table S1.
Transmission ratios of NFCas9-2 transgene from F1 Tg/+ males or F1 Tg/+females to backcrossed N2 offspring.

Types of crosses ♀ ♂	No. of N2 examined	Transmission ratio observed in N2 ♀ Tg/+ : ♀ +/+: ♂ Tg/+: ♂+/+	Transmission ratio expected in N2 ♀ Tg/+ : ♀ +/+: ♂ Tg/+: ♂+/+	Chi-square value	P
+/+ x F1 Tg/+	68	19: 16: 18: 15	17: 17: 17: 17	6.23	0.90
F1 Tg/+ x +/+	16	5: 4: 3: 4	4: 4: 4: 4	6.73	0.91

+/+ BDF1, B6

Table S2.
Transmission ratios of NFCas9-2 transgene from F1 Tg/+ mice to intercross N2 offspring.

Types of crosses ♀ ♂	No. of N2 examined	Transmission ratio* observed in N2 Tg/Tg : Tg/+ : +/+	Transmission ratio expected in N2 Tg/Tg* : Tg/+ : +/+	Chi-square value	P
F1 Tg/+ x F1 Tg/+	22	5: 11: 6	5.5: 11: 5.5	6.23	0.96

* Zygosity of N2 mice were identified by the method of Sakurai et al., (2008)[38]

Table S3.
The rate of generation of multiple indel mutant alleles in blastocysts derived from wild zygotes (+/+ female x +/+ male) injected with both *Cas9* mRNA and nine different of gRNAs.

Concentration of injected RNAs: *Cas9* mRNA ng/μ l	Concentration of injected RNAs: Mixed 9 gRNAs ng/μ l	No. alive/ no. injected fertilized eggs (%)	No. blastocyst/ no. alive fertilized eggs (%)	No. +/+ blastocysts analyzed	No. blastocysts having indel mutant alleles: 0-5	6-7	8	9
100	100	21/25 (84)	4/21 (19)	4	1	0	1	2
100	200	21/25 (84)	3/21 (14)	3	0	0	2	1
100	300	27/28 (96)	3/27 (11)	3	0	0	2	1
200	100	21/25 (84)	1/21 (5)	1	0	0	0	1
200	200	22/25 (88)	1/22 (5)	1	0	0	1	0
0	0	14/14 (100)[1]	12/14 (86)	ND	ND	ND	ND	ND

1. Cas9 mRNAs and 9 gRNAs were not injected into fertilized eggs.
ND= not determined.

Table S4. gRNA sequences.

ID	Sequences (5' -> 3')	Location	GeneBank#	Notes
Alb-gRNA	CGCAGATGACAGGGTAAGGAAGG	near *Alb* exon 7	NM009654	Figures 4, 6 and Table S3
Amy-gRNA	ATATGTATTCGATCCCACGTTGG	*Amy* exon 2	NM010491	Figure 4 and Table S3
Crlr-gRNA	ATGTGATCCGTTGGCAACTTAGG	*Crlr* exon 6	NM018782	Figure 4 and Table S3
Et-1-gRNA	GCGCGTCGTACCGTATGGACTGG	*Et-1* exon 3	NM010104	Figure 4 and Table S3
Ggat1-gRNA	GAGAAAATAATGAATGTCAAAGG	*Ggta1* exon 4	NM001145821	Figures 4, 5 and Table S3
Hprt-gRNA	GGGACTGCGGGTCGGCATGACGG	*Hprt* exon 1	NM013556	Figure 4 and Table S3
Imdn-gRNA	GGCTGCCAGACAACGTGCCGGGG	*Imdn* exon 2	NM182928	Figure 4 and Table S3
Klf5-gRNA	GGTCAGCACCCGCGTGGGCATGG	*Klf5* exon 1	NM009769	Figure 4 and Table S3
R1-gRNA	GCTCTGCTTGCCATGGCCCCGGG	*Ramp1*exon 1	NM001168392	Figures 4, S5, S6 and Table S3
R2-gRNA	GGTGAGCACCGTGTCGGGCCCGG	near *Ramp2* exon 1	NM019444	Figures 2, 4, S5, S6 and Table S3

The PAM sequence is shown in red. The genomic locations of these gRNA sequences are shown in Figs. 2c, 5b, 6b and S7.

Table S5. Primers used in this study.

ID	sequences (5' -> 3')
Amy-1S	CGGTGAAGGGTGTTGTGTGA
Amy-1A	ACACAGTCATCAAGCACAAGC
Alb-7S	CTAACGCCTGCTGGTGGTCACAGT
Alb-7A	TGTCTAGCCCCTGCTTCTCT
CAG1620-S	GCTCTAGAGCCTCTGCTAACCATGT
CAGGS-A	GCGGAACTCCATATATGGGCTATGAACTAATG
Cas9 215-A	CGCCGTGCTGTTCTTTTGAGC
Cas9ATG-S	GCCACCATGGACAAGAAGTACTCCATTG
Cas9Cla-A	CCATCGATTCACACCTTCCTCTTCTTCT
Cas9E-S	AAGCGAATTCTCCAAAAGAGTGAT
Crlr-5S	AGAGTTTGCTGAGGCAGGTC
Crlr-5A	CCAGCCCATGTCTCTTCCTA
Et-1-1S	TGGTGTGGCTGTAGTCCTTC
Et-1-1A	CAAGTCAAAGGGGCCTCCAC
Ggta1-S	TCAACCACACAGCTGTTTCTC
Ggta1-A	CTGGCACCAGTCACAGGGAATG
Gtpbp10-S	GTTGTTATAATCTTCTAACAG
Gtpbp10-A	CTGGGTGGAACAATTGATCGT
Hprt-1S	TGAGCCATTGCTGAGGCG
Hprt-1A	CGCGCCTGATCCTTCCTG
Hprt-SF	TGTTGGATACAGGCCAGACTTTGTT
Hprt-SR	TCTTAGGCTTTGTATTTGGCTTTTC
Imdn-1S	ACGCAGGTACCAACCAATCT
Imdn-1A	CCCGGGTTCTTCCCTTATGT
Klf5-1S	GGTACGCGCTCTCTTAGGTT
Klf5-1A	GCGTGTTTCAGATCGTCTCC
Ramp1-2S	GTCAGAGCGAGGTGCTGAGT
Ramp1-2A	CCCATCACCACTCTACTGTCTTG
Ramp1-SF	GCACTGGTGGTCTGGAGGA
Ramp1-SR	CCCTCATCACCTGGGATACCT
Ramp2-SF	TGTGCCTCCCTCCGCTGTT
Ramp2-SR	TTACTCCTCCACACCACAAGA
Ramp2-5S	CCGAGCTGGAAGCGAGAG
Ramp2-6A	GACCTCTCCGTCCGGTCT
Ramp3-S	TCGAATTCATCTTAGTTGGCCATGAAGAC
Ramp3-A	TCGAATTCACAGCAGCCGATCAGTGTGCTTG
Sp#2	GTGGCTGAATGAGACTGGTGTCGAC
Sp-Bottom	CGAAGAGTAACCGTTGCTAGGAGAGACCGTGGCTGAATGAGACT-<br>-GGTGTCGACACTAGTGG.
Sp-EV Top	CCACTAGTGTCGACACCAGTCTCTAATTTTTTTTTTCAAAAAAA

Table S6. Primers used for off-target analysis in this study.

Gene	ID	Sequences (5' -> 3')
For Ramp1 gene		
Ccdc3(NM_028804)	OfftR1-11	TATATCTGTTTTCCAACCCCAGCTC
	OfftR1-12	TGGGGTACACGTAGACCTTTTATGA
Trappc13(NM_001093760)	OfftR1-21	TCTCTGGCTGAATTGTTTTACACAGG
	OfftR1-22	AAAGCAGTCAGCGAACCTTTTTG
Krt5(NM_027011)	OfftR1-31	ATGTCTCGCCAGTCCAGTGTGTC
	OfftR1-32	CACCACCAAAACCAAATCCACTG
For Ramp2 gene		
Chpf(NM_001001565)	OfftR2-11	ACTCAGACGTCTTCGCACCTGTC
	OfftR2-12	CGCCACATAGTCGGAGTTGTAGA
B3gat1(NM_029792)	OfftR2-21	CACCTCTGCAGATGAGGGAAGTG
	OfftR2-22	GGGCATCACCTCGCAGCTTATAG
Sema6b(NM_001130456)	OfftR2-31	CGCCTTTGACATGAACCAAGTG
	OfftR2-32	ACTCACTGTAGCCTCGGGACCTC

Table S7. Ramp1 and Ramp2 Off-target analysis related to expreriment 1 of Figure S6.

Gene	Chr.	Sequences*	No. mismatches	Mutation frequency (Mutant/Total)
For Ramp1				
Ccdc3(NM_028804)	2	GCGCAGCTTGCCATGGCTCCGAG	3	0/10
Trappc13(NM_001093760)	13	GACCTGCTTGCCAAGGCCCCTAG	3	0/10
Krt5(NM_027011)	15	GCTCCGGCTGCCATAGCCCCCGG	4	0/10
For Ramp2				
Chpf(NM_001001565)	1	TGTGAGTACCGTGTCTGGCCCGG	3	0/10
B3gat1(NM_029792)	9	GGTGGGCAGCGTGTCGGACCAGG	3	0/10
Sema6b(NM_001130456)	17	GGTCAGCTCAGTGTCGGGTCAGG	4	0/10

* Sequences of off-target candidates were determined by the CRISPR Designe tool (http://crispr.mit.edu). The presence of indel mutations was analyzed by sequencing uisng primers shown in Table S6.

Table S8. Summary of the results of Ramp2 gene off-target analysis.

Gene	Chr.	Sequences *	No. mismatches	Mutation frequency ** in blastocysts (no. mutated embryos/no. total embryos tested)		
				maCas9		Wild-type
				+/+	Tg/+	+/+
Chpf(NM_001001565)	1	TGTGAGTACCGTGTCTGGCCCGG	3	0/10	0/10	0/10
B3gat1(NM_029792)	9	GGTGGGCAGCGTGTCGGACCAGG	3	0/10	0/10	0/10
Sema6b(NM_001130456)	17	GGTCAGCTCAGTGTCGGGTCAGG	4	0/10	0/10	0/10

* Sequences of off-target candidates were determined using the CRISPR designe tool (http://crispr.mit.edu).

** Ten blastocysts with each genotypes were randomly collected and analyzed for the presence of indel mutations using sequencing with primers shown in Table S6.
